# Cardio-kidney-metabolic (CKM) framework: A nephrologist’s perspective

**DOI:** 10.20945/2359-4292-2025-0405

**Published:** 2025-11-24

**Authors:** Ana Flavia Moura, Roberto Pecoits-Filho

**Affiliations:** 1 Escola Bahiana de Medicina e Saúde Pública, Departamento de Medicina Interna, Salvador, BA, Brasil; 2 ClinEpi Program, Arbor Research Collaborative for Health, Ann Arbor, MI, USA; 3 Division of Nephrology, University of Michigan, Ann Arbor, MI, USA; 4 Escola de Medicina, Pontifícia Universidade Católica do Paraná, Curitiba, PR, Brasil

**Keywords:** Cardio-kidney-metabolic syndrome, chronic kidney disease, obesity, diabetes, cardiovascular disease

## Abstract

The recently proposed cardio-kidney-metabolic (CKM) framework underscores the interconnected nature of cardiovascular, renal, and metabolic diseases and represents an important step toward preventive, integrated care. However, its application in kidney care remains limited and dependent on additional supportive evidence. Chronic kidney disease (CKD) is often underrecognized in cardiovascular risk models and receives delayed attention within the CKM pathway. Nephrologists face unique challenges - including workforce shortages, late referrals, and fragmented care systems - particularly in lowand middleincome countries. Early detection is further hindered by the lack of CKD-specific risk assessment tools and limited access to essential diagnostics and therapies. Real-world data from global and national studies highlight substantial implementation gaps, suboptimal outcomes, and the heavy economic burden of delayed CKD management. Importantly, as emphasized by the American Heart Association, implementation of the CKM approach is still under construction and must remain data-driven, ensuring that strategies are grounded in robust evidence. This review offers a nephrology-oriented perspective on the CKM framework, emphasizing the bidirectional relationship between CKD and other CKM components, the prognostic implications of delayed diagnosis, and the need for improved multidisciplinary coordination.

## INTRODUCTION

The cardio-kidney-metabolic (CKM) framework, recently proposed by the American Heart Association, emphasizes the interplay between cardiovascular, renal, and metabolic diseases in determining survival and quality of life (^1^). The impact of obesity, diabetes, kidney disease, and cardiovascular disease on mortality is well documented, with cardiovascular disease remaining the leading global cause of death (^2^-^6^). Individuals with a BMI > 25 kg/m^2^ have a 30% higher mortality risk for every 5 kg/m^2^ increment (^3^).

Patients with diabetes or CKD also face higher mortality (^4^,^5^), and those with eGFR < 30 mL/min/1.73 m^2^ live on average 20 years less than individuals with preserved kidney function (≥60 mL/min/1.73 m^2^) (^4^).

The relationship between CKM syndrome and CKD is bidirectional: CKD predisposes to hypertension, insulin resistance, and dyslipidemia, yet remains underrepresented in cardiovascular risk models despite its profound impact on outcomes (^7^,^8^). This underrecognition dilutes its clinical significance and delays targeted interventions, even though CKD independently increases cardiovascular risk and frequently coexists with diabetes, hypertension, and obesity.

While the CKM framework promotes integrated, multidisciplinary care, it does not fully capture the practical challenges of nephrology - particularly in lowand middle-income countries (LMICs), where workforce shortages, limited resources, and fragmented systems hinder early detection and intervention (^9^). In many LMICs, nephrologists are concentrated in tertiary centers or dialysis clinics, leaving large populations without specialist access (^10^). As a result, serum creatinine and albumin-tocreatinine ratio (UACR) testing - essential for CKD detection - are often underused due to infrastructure and training gaps (^11^).

These limitations have measurable consequences. REVEAL-CKD showed that patients with unrecorded stage 3 CKD experienced delayed initiation of diseasemodifying therapies and inadequate risk stratification (^12^). The Brazilian CHECK-CKD study similarly highlighted systemic deficiencies in the interpretation of kidney function at the primary care level (^13^). With dialysis needs in Brazil projected to increase by 170.8% by 2032 (^14^), earlier nephrology engagement is critical.

This review provides a nephrology-oriented perspective on the CKM framework, emphasizing the importance of timely referral, access to kidneyprotective therapies, and embedding nephrology within multidisciplinary teams to bridge current implementation gaps.

## THE INTERSECTION OF CARDIO-KIDNEYMETABOLIC HEALTH: A NEPHROLOGYORIENTED PERSPECTIVE

The intersection between cardiovascular, renal, and metabolic systems is of growing relevance in nephrology. This is not only due to overlapping risk factors but also to the tightly intertwined pathophysiological mechanisms that drive CKD progression and its complications. Understanding these interactions is crucial for the modern nephrologist, as clinical and therapeutic decisions frequently require an integrated approach to managing these domains.

### Core components of the CKM axis

The CKM framework includes a wide range of conditions distributed across three major domains. The cardiovascular domain encompasses hypertension, atherosclerotic vascular disease, ischemic heart disease, atrial fibrillation, heart failure (HF), and non-hemorrhagic cerebrovascular disease. The renal domain includes primary kidney diseases - such as glomerulopathies, nephrolithiasis, and polycystic kidney disease - as well as secondary CKD and end-stage kidney disease (ESKD). Metabolic disturbances within the CKM spectrum include central obesity, insulin resistance, dyslipidemia, type 2 diabetes mellitus (T2DM), non-alcoholic fatty liver disease, and polycystic ovary syndrome (^15^).

While the CKM framework appropriately highlights the intersection of cardiometabolic drivers such as obesity, diabetes, and hypertension with kidney disease, it risks underrepresenting a broad spectrum of primary renal disorders that do not fit neatly within this paradigm. Glomerulonephrites, hereditary cystic diseases, and obstructive urological disorders account for a substantial proportion of chronic kidney disease worldwide and are associated with unique trajectories and outcomes that cannot be fully explained by cardiometabolic clustering. Failure to integrate these conditions more explicitly within the CKM framework may inadvertently marginalize patients whose kidney disease arises from non-cardiometabolic pathways, thus limiting the generalizability of the model across the full spectrum of nephrology practice.

### Hypertension in chronic kidney disease

Hypertension is the second most common cause of ESKD worldwide, and CKD is three times more prevalent among individuals with hypertension compared to the general population (^16^). Most hypertension-related renal injuries are attributed to the loss of autoregulatory mechanisms within the renal microvasculature, resulting in arteriolar wall hypertrophy, activation of endothelin-1 signaling pathways, and hyperactivation of both the renin-angiotensinaldosterone system (RAAS) and the sympathetic nervous system (SNS). These processes lead to efferent arteriolar vasoconstriction, sodium and water retention, and the initiation of pro-fibrotic and proinflammatory cascades (^17^). When sustained, these disturbances cause hemodynamic and histopathological alterations that impair glomerular function. Over the medium to long term, progressive deterioration of kidney function further amplifies these pathophysiological mechanisms, particularly RAAS hyperactivation, intensifying pro-inflammatory and pro-fibrotic responses and contributing to vascular dysfunction (^17^).

## INSULIN RESISTANCE AND CKD

Peripheral insulin resistance is frequently observed in CKD and inversely correlates with eGFR, even among non-diabetic individuals (^18^). This multifactorial condition is driven by chronic inflammation, RAAS activation, uremic toxins, metabolic acidosis, gut microbiota dysregulation, and oxidative stress - hallmarks of CKD - which collectively downregulate the GLUT4 glucose transporter responsible for insulin-dependent cellular glucose uptake (^19^,^20^). Diabetes is reported in approximately 28% of CKD patients and remains the leading global cause of ESKD (^19^,^20^).

## OBESITY AND THE KIDNEY

Obesity is a central feature of CKM syndrome and is increasingly prevalent both in the general population and among CKD patients (^21^,^22^). Visceral adiposity induces a lipotoxic and pro-inflammatory state that promotes oxidative stress, endothelial dysfunction, and atherosclerosis (^21^,^22^). Moreover, visceral fat may exert mechanical pressure on the glomeruli, enhancing sodium reabsorption in the loop of Henle, activating tubuloglomerular feedback, and stimulating the RAAS axis-all contributing to hemodynamic alterations and kidney injury (^22^-^24^). Adipocytes also act as aldosterone secretagogues, further elevating the risk of hypertension and subsequent CKD progression (^25^).

Although obesity is a recognized risk factor for CKD onset and progression, it paradoxically appears to confer a survival benefit in dialysis patients - a phenomenon known as the “obesity paradox” (^26^). The mechanisms underlying this observation remain unclear and are under active investigation.

## DYSLIPIDEMIA IN CKD AND ESKD

Dyslipidemia, particularly hypertriglyceridemia, is frequently associated with CKD and ESKD (^27^). The pro-inflammatory state in CKD exacerbates insulin resistance and impairs lipoprotein lipase (LPL) activity through the overproduction of inhibitory molecules and the development of secondary hyperparathyroidism (^27^). These alterations reduce the hydrolysis of chylomicrons and very-low-density lipoproteins (VLDL). Dialysis therapies - both hemodialysis and peritoneal dialysis - can further exacerbate dyslipidemia by intensifying systemic inflammation and promoting protein loss (^27^,^28^). Additionally, patients with nephrotic syndrome often present with pronounced dyslipidemia, especially elevated low-density lipoprotein (LDL) cholesterol (^29^).

## CKM SYNDROME AS A RISK FACTOR FOR KIDNEY DISEASE

Beyond the factors described above, CKM syndrome itself has been independently associated with increased risk of developing CKD (^30^). Studies have shown that individuals with CKM syndrome have approximately a 1.5-fold higher risk of CKD compared to the general population (^31^). This risk rises progressively with the accumulation of CKM components- namely dyslipidemia, hyperglycemia, insulin resistance, obesity, and hypertension (^31^,^32^). Although the exact pathophysiological mechanisms remain unclear, they are thought to involve oxidative stress, endothelial dysfunction, glomerular injury, and pro-fibrotic signaling.

## PROGNOSTIC IMPLICATIONS OF CKM SYNDROME

There is consistent evidence that CKM syndrome is linked to increased mortality and adverse cardiovascular outcomes (^5^,^15^). However, it remains unclear whether CKM syndrome continues to act as an independent risk factor after adjusting for the individual comorbidities that define it. Emerging studies suggest that CKM syndrome may indeed serve as an independent predictor, even after controlling for cardiovascular, renal, and metabolic variables (^5^,^15^,^33^). Additional studies are needed to confirm and refine these findings. **Table 1** summarizes the primary mechanisms linking CKM syndrome and CKD.

**Table 1:** CKM mechanisms and Nephrology relevance

Domain	Pathophysiological Mechanism	Relevance to Nephrology
Cardiovascular	Systemic arterial hypertension	Second leading global cause of ESKD; key therapeutic target in CKD
Cardiovascular	Endothelial dysfunction and atherosclerosis	Increases cardiovascular risk in CKD patients
Cardiovascular	Sympathetic nervous system and RAAS activation	Contributes to renal fibrosis and proteinuria
Renal	Loss of renal microvascular autoregulation	Central mechanism in hypertensive CKD progression
Renal	Efferent arteriolar vasoconstriction and glomerular inflammation	Leads to hemodynamic changes that reduce GFR
Renal	Sodium and water retention	Promotes volume overload and resistant hypertension
Metabolic	Insulin resistance and hyperglycemia	Leading global cause of CKD; worsens tubular injury
Metabolic	Chronic inflammation and oxidative stress	Present across CKD stages; aggravates renal injury
Metabolic	Dyslipidemia (hypertriglyceridemia, increased LDL)	Associated with CKD progression and increased cardiovascular risk
Metabolic	Visceral adiposity and RAAS activation	Promotes glomerular injury and contributes to hypertension

**Figure 1:**
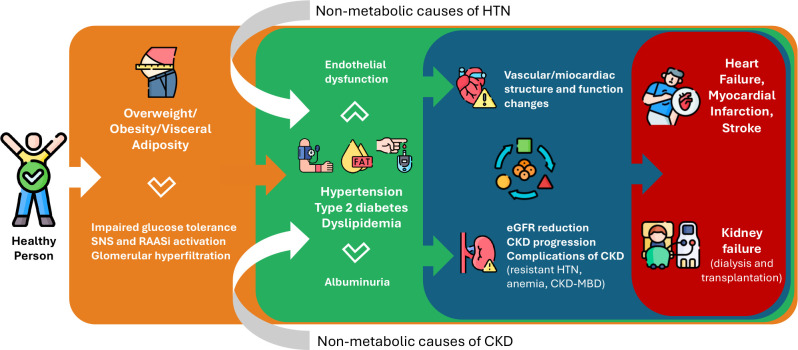
Mechanistic interplay between CKM syndrome and CKD progression.

SNS: sympatic nervous system; RAASi: renin-angiotensin-aldosterone system inhibitors; CKD: chronic kidney disease; CV: cardiovascular; eGFR: estimated glomerular filtration rate; HTN: hypertension; MBD: mineral bone disease

Figure 1 illustrates the bidirectional interactions between cardiovascular, renal, and metabolic domains, highlighting the pathophysiological mechanisms that drive CKM syndrome and its clinical consequences.

## CURRENT GAPS IN CKM FROM A NEPHROLOGY PERSPECTIVE

Despite the clear association between CKM syndrome and kidney disease, nephrology assessment and early identification of renal risk are not consistently addressed in clinical practice. Many current guidelines tend to emphasize individual risk stratification and the isolated management of cardiovascular, renal, and metabolic diseases, while offering limited guidance on integrated care strategies. This fragmented approach overlooks the importance of multidisciplinary coordination aimed at aligning diverse healthcare professionals toward a shared goal in the management of CKM patients (^34^).

An important limitation of the current AHA conceptualization lies in its outcome prioritization. The Presidential Advisory places considerable emphasis on cardiovascular morbidity and mortality, yet renal failure itself constitutes a major and devastating outcome, particularly for patients who progress to kidney replacement therapy. End-stage kidney disease carries not only profound prognostic and economic consequences but also a heavy social burden, often exceeding that of cardiovascular complications in certain regions and populations (^14^). By minimizing kidney failure as a primary endpoint, the framework risks perpetuating a cardiovascular-centric bias and diluting the urgency of interventions aimed at preventing progression of kidney disease.

Real-world evidence from the REVEAL-CKD study highlighted that a lack of reporting and delayed diagnosis are frequent and consequential: approximately 61% of patients with stage 3 CKD were undiagnosed (^12^), and a delay of just one year was associated with a 63% higher risk of kidney failure, a 40% higher risk of CKD progression, and an 8% increase in cardiovascular events. These findings illustrate how delayed detection and referral may significantly worsen outcomes (^12^).

Moreover, many clinical trials exclude patients with CKD, particularly those with ESKD, those undergoing dialysis, or kidney transplant recipients. As a result, there is a significant lack of prospective studies specifically addressing these population (^21^). Consequently, clinicians are often required to extrapolate recommendations and outcomes derived from studies conducted in other patient groups to individuals with kidney disease, which may not fully reflect their unique clinical context.

Another significant challenge lies in the diagnosis of kidney injury associated with CKM syndrome, as no well-defined diagnostic or histopathological criteria currently exist. Certain biomarkers, such as growth differentiation factor-11 (GDF-11), GDF-15, and urinary A-megalin, have shown correlation with CKM syndrome, yet they remain unvalidated for routine clinical use (^35^-^37^). Concurrently, in some countries, there is a lack of adequate training among primary care physicians (PCPs) in the interpretation of essential laboratory tests for CKD screening - such as UACR and serum creatinine measurement - thereby limiting early diagnosis and risk stratification (^34^). Furthermore, access to essential medications for the conservative management of CKD, T2DM and cardiovascular conditions remains restricted in many regions, undermining optimal disease control.

Although risk stratification is a highly emphasized aspect in CKM syndrome guidelines, there remains a lack of risk assessment tools that are appropriately tailored to patients with CKD or CKM syndrome. Most validation studies for such tools either exclude individuals with CKD or fail to incorporate markers of kidney function and structural kidney damage - such as UACR - as part of the risk criteria. The AHA Presidential Advisory on CKM syndrome acknowledges that most currently available cardiovascular risk calculators are suboptimal for use in patients with CKD and therefore refrains from endorsing any specific tool for this population (^6^). Instead, the advisory recommends stratifying CKM syndrome based on its core risk determinants, with particular emphasis on atherosclerotic cardiovascular disease (ASCVD) risk, the presence of T2DM, and CKD (^6^).

This gap carries important clinical implications. Using risk scores not validated in CKD populations may result in under-recognition of cardiovascular risk, especially in patients with significant albuminuria or reduced eGFR. Consequently, this can lead to undertreatment, delayed initiation of disease-modifying therapies, or inappropriate reassurance.

For instance, data from the CKDopps study revealed that fewer than 40% of non-dialysis CKD patients with hemoglobin levels below 10 g/dL received anemia treatment over a 12-month period. Similarly, iron supplementation was infrequently prescribed even among patients with iron deficiency. These findings underscore the risk of clinical inertia or misclassification when standardized nephrologyinformed tools are not used (^38^).

Following the publication of the AHA Presidential Advisory on CKM syndrome, AHA introduced a novel cardiovascular risk stratification tool - the PREVENT score (^39^). This risk calculator incorporates renal, metabolic, and cardiovascular parameters, making it more suitable for individuals with CKD and CKM syndrome. Additionally, it expands the eligible population by including adults as young as 30 years of age, thereby supporting earlier intervention and lifelong risk assessment (^39^).

However, despite its strengths, the PREVENT score still does not incorporate risk of kidney failure (KF), nor does it integrate CKD-specific factors such as anemia, mineral and bone disorder (CKD-MBD), or electrolyte disturbances. This limits its utility for nephrologists aiming to manage cardio-kidney risk comprehensively.

Finally, modeling from the IMPACT-CKD simulation study provided compelling evidence on the consequences of delayed CKD identification and the potential benefits of early intervention in eight countries (^14^). In Brazil, the simulation projected a 6.9% increase in the number of patients with stage 3-5 CKD over the next 10 years, resulting in approximately 370,000 individuals requiring dialysis by 2032 (^14^). Furthermore, it forecasted a 100.6% rise in CKDrelated cardiovascular events and a 67.8% increase in mortality during the same period (^14^). Annual dialysisrelated costs were estimated at around US$2.7 billion, representing 25.7% of Brazil’s total healthcare budget across the decade (2023-2032) (^14^). These findings underscore the urgent need for early, coordinated CKD care models - including nephrology involvement - to mitigate clinical, enviromental, social, and economic burdens in the Brazilian context (^14^).

## LEVERAGING THE CKM FRAMEWORK TO ADVANCE KIDNEY CARE

### Risk stratification and implementation gaps

The AHA recommends that patients with CKM syndrome be stratified according to one of the five risk stages proposed in its Presidential Advisory (^6^). While this staging framework offers a clear path for tailoring interventions across different phases of cardiometabolic disease, its real-world application is challenged by significant gaps in implementation. Studies such as CKDopps and the CHECK and SEARCH initiatives have highlighted considerable variability in adherence to screening and referral guidelines, especially for CKD, which is a central component of CKM syndrome (^13^,^38^,^40^).

Although albuminuria testing is critical for risk stratification, it remains vastly underutilized in clinical practice. For instance, data from the CKDopps study reveal that albuminuria is routinely measured in fewer than 30% of patients with diabetes or hypertension in some health systems, including in Brazil, despite clear recommendations for its use in early CKD detection and cardiovascular risk prediction (^11^). Similarly, the CHECK study demonstrated that systemic barriers - including limited access to diagnostic tools, fragmented care pathways, and undertrained primary care teams - impede consistent implementation of evidence-based CKD screening practices (^13^).

Findings from the SEARCH study - a nationwide CKD screening campaign in Brazil - revealed that approximately 20% of high-risk individuals with diabetes and/or hypertension had previously undiagnosed CKD, with over 11% presenting albuminuria > 30 mg/g (^40^). Alarmingly, fewer than 9% of patients with moderate or severe albuminuria (A2/A3) had a repeat ACR measured within two years following diagnosis, despite receiving formal documentation of their CKD stage (^40^). These findings highlight the persistent underuse of albuminuria testing in real-world practice, even after disease recognition, and underscore a missed opportunity for risk stratification and early intervention in patients with cardiometabolic risk profiles (^40^).

These deficiencies have meaningful consequences. Modeling from the IMPACT-CKD study projected that by 2032, CKD prevalence would range between 11.7% and 16.5% in eight countries, increasing in seven of them (^14^). In Brazil, the number of patients requiring dialysis is expected to rise by 170.8% between 2022 and 2032 (^14^). These findings reinforce that failing to act during earlier CKM stages - especially stages 1 and 2 - carries high clinical and economic penalties.

Moreover, outcomes remain poor even for patients who do receive nephrology care in non-high-income countries. A recent multinational study evaluating over 6,000 patients under nephrology follow-up found that mortality and progression to kidney failure were significantly higher in low-resource settings, driven by late referral, limited access to essential therapies, and underutilization of evidence-based risk stratification tools such as albuminuria testing. These findings further emphasize the need for earlier engagement of nephrology services within the CKM framework, particularly in health systems facing structural limitations (^41^).

To address these issues, it is essential to integrate guideline recommendations into feasible, scalable care models. Routine inclusion of both serum creatinine and UACR from Stage 0 onward can improve early detection. Moreover, referral patterns to nephrologists must shift from a reactive to a proactive approach. The AHA and KDIGO both support earlier referral, particularly for patients at Stage G3aA3 or G3bA2, where intervention has the potential to significantly alter the disease course (^6^,^7^). Yet, as CKDopps data show, most referrals still occur at advanced stages, often when irreversible organ damage has already occurred (^11^).

Therefore, leveraging the CKM framework effectively requires not only clinical awareness but also health system reform. This includes training primary care providers to identify CKD risk early, embedding nephrology input in multidisciplinary teams, and ensuring equitable access to essential diagnostics and therapies. By addressing these implementation barriers head-on, it becomes possible to transform CKM staging from a conceptual model into a population-level strategy for improving kidney and cardiovascular outcomes.

## BARRIERS TO IMPLEMENTATION IN NEPHROLOGY PRACTICE

**Figure 2:**
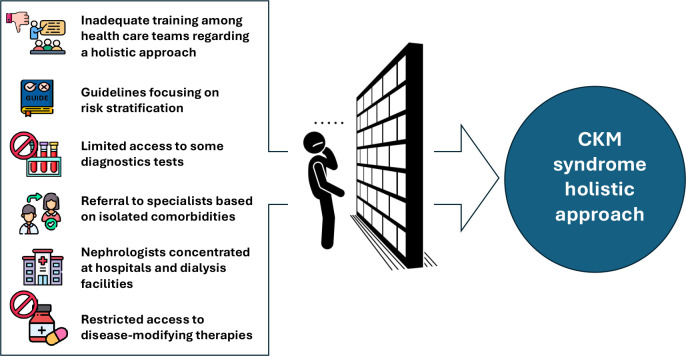
Key barriers to the implementation of holistic care in CKM syndrome.

Despite the high prevalence and clinical complexity of CKM syndrome, the implementation of integrated care remains suboptimal. While most major clinical guidelines provide robust frameworks for risk stratification and syndromic classification, they often fall short in translating these concepts into actionable care models. As a result, multidisciplinary coordination across cardiology, nephrology, and endocrinology is frequently fragmented, undermining the delivery of patient-centered, holistic care (^7^,^12^). **Figure 2** summarize main barriers to CKM holistic care implementation.

### Health system barriers

Many healthcare systems, especially in lowand middle-income countries, remain structurally oriented toward acute care and are ill-equipped to manage chronic, multisystem diseases like CKM syndrome. Diagnostic limitations are common: access to essential tests such as serum creatinine, UACR, and natriuretic peptides is often restricted or delayed, impairing timely identification of kidney, cardiac, and metabolic abnormalities. In Brazil, for example, fewer than 30% of high-risk patients undergo albuminuria testing despite guideline recommendations (^11^). Specialist referral is also frequently delayed due to diagnostic uncertainty, limited subspecialty availability, and centralized nephrology services located primarily in tertiary or dialysis-based settings.

Compounding these issues, some public health policies recommend delayed specialist referral (*e.g.*, to nephrologists only when eGFR falls below 30 mL/min/1.73 m^2^), largely to prevent overwhelming limited specialist capacity. However, this approach inadvertently leads to late-stage diagnoses and missed opportunities for early therapeutic intervention.

In response to the rising burden of CKD in low-resource settings, the World Health Organization (WHO recognized CKD as a growing public health priority through the first declaration dedicated to kidney disease. The WHO highlighted the need to strengthen early detection programs, expand access to essential diagnostic and therapeutic tools, and improve care coordination - especially for populations at risk due to environmental, occupational, and cardiometabolic factors (^42^). This declaration reinforces the urgency of developing national strategies that address CKD not only as a renal issue but as a systemic and societal challenge.

### Educational and training barriers

Another key barrier lies in the undertraining of primary care professionals in CKD recognition and management. The CHECK study demonstrated that even when guidelines are well established, inadequate professional training and poor integration of nephrology expertise into primary care settings result in missed opportunities for early diagnosis and intervention (^13^). This educational gap contributes to late referrals, suboptimal risk stratification, and delayed initiation of kidney-protective therapies.

Even in specialized centers such as dialysis clinics, there is often a lack of familiarity with the broader CKM framework. In many cases, patients undergoing dialysis receive nephrology care that is narrowly focused on kidney replacement therapy, with limited attention to concurrent cardiovascular or metabolic conditions. This siloed care model perpetuates underrecognition of heart failure and atherosclerotic disease, which are common but often overlooked in the dialysis population (^41^). Given that dialysis teams frequently serve as the primary - if not sole - source of specialist care for these patients, equipping them to address all aspects of CKM syndrome is imperative.

### Therapeutic access barriers

Access to key medications for CKD, T2DM, HF, and obesity - such as SGLT2 inhibitors, GLP-1 receptor agonists, and nonsteroidal mineralocorticoid receptor antagonists - remains limited in many healthcare systems. Cost, regulatory constraints, and supply chain limitations have all contributed to low uptake, particularly outside of urban tertiary centers. Although progress has been made in expanding coverage for select drug classes, such improvements remain uneven and insufficient to meet population-level needs. Without consistent access to these disease-modifying therapies, even timely diagnosis is unlikely to yield optimal outcomes.

## FUTURE DIRECTIONS AND CALL TO ACTION

The multisystem nature of CKM syndrome presents complex challenges that demand truly integrated, multidisciplinary care (^43^). Telemedicine has emerged as a promising tool to strengthen communication among specialists, enabling more frequent and even real-time discussions that support coordinated management (^44^,^45^). Alternating teleconsultations with in-person visits can also facilitate closer follow-up, particularly for patients in rural areas or in countries with vast geographic distances and limited access to specialized services (^45^).

Future refinements of the CKM framework should therefore adopt a more balanced approach that equally acknowledges renal-specific outcomes alongside cardiovascular events. This would require explicit inclusion of kidney failure prevention as a co-primary goal, as recommended in recent KDIGO guidelines on CKD management (^7^). Moreover, a more comprehensive taxonomy of kidney diseases - including immune-mediated, genetic, and structural disorders - would strengthen the relevance of the framework for nephrology, ensuring that it reflects the true heterogeneity of kidney disease across global populations.

The American Heart Association has launched several initiatives to advance CKM care. The *Get With The Guidelines* (GWTG) program systematically collects clinical data, monitors adherence to evidence-based practices, and benchmarks institutional performance, fostering a learning health system (^6^). More recently, the CKM Centers of Excellence initiative was introduced to establish dedicated institutions that integrate cardiovascular, renal, and metabolic care under one framework. These centers are designed to model best practices, promote research, and serve as hubs for multidisciplinary training. Importantly, the AHA has emphasized that implementation of the CKM framework is still under construction and must remain data-driven, ensuring that best practices are guided by real-world evidence. At present, both GWTG and the Centers of Excellence remain U.S.-based initiatives, highlighting the need for global expansion.

International efforts are also gaining momentum. The International Society of Nephrology (ISN) will convene an expert forum on CKM syndrome in October 2025, bringing together leaders from nephrology, cardiology, endocrinology, and health policy. This forum will generate a consensus document outlining key messages from the group, expected to be published later this year. Such initiatives represent important steps toward building a coordinated global approach, but must also remain anchored in robust, data-driven strategies to ensure effectiveness and sustainability.

Bridging the gap between evidence and practice requires guidelines that provide real-world implementation strategies, expanded reimbursement criteria, and broader access to diagnostics and therapies. Workforce investment is equally important: developing career pathways and improving conditions for primary and secondary care providers can help attract specialists to non-tertiary settings, thereby expanding access for patients living with CKM syndrome. Continuing medical education and holistic patient assessment must also become routine across specialties. Finally, the systematic inclusion of CKD and ESKD patients in clinical trials is essential to generate evidence that reflects the populations most affected by CKM syndrome.

## CONCLUSION

The CKM framework represents a critical step toward integrated, patient-centered care across cardiovascular, renal, and metabolic domains. Yet implementation remains limited by late recognition of CKD, fragmented health systems, and insufficient access to diagnostics and therapies. To realize its potential, the CKM model must embed nephrology expertise earlier in the care pathway, promote proactive screening and referral, and ensure equitable access to evidence-based treatments.

Initiatives such as the American Heart Association’s *Get With The Guidelines* program and the CKM Centers of Excellence provide important models for advancing multidisciplinary care. Crucially, the AHA has stressed that implementation of the CKM approach is still in development and must remain data-driven - a principle that should guide all future actions. The upcoming International Society of Nephrology (ISN) expert forum on CKM syndrome in October 2025 represents another milestone, bringing together global experts to define priorities and publish a consensus document later this year.

Expanding these initiatives globally, while keeping data at the center of implementation, will be essential to address the rising burden of CKM-related disease. Ultimately, a coordinated international strategy - combining high-quality data, practical implementation tools, and cross-specialty collaboration - will be decisive in transforming the CKM framework from concept to practice and in improving outcomes for patients worldwide.

## Data Availability

datasets related to this article will be available upon request to the corresponding author.
